# How frequently are predicted peptides actually recognized by CD8 cells?

**DOI:** 10.1186/2051-1426-1-S1-P111

**Published:** 2013-11-07

**Authors:** Srividya Sundararaman, Ioana Moldovan, Oleg Targoni, Wenji Zhang, Paul V Lehmann

**Affiliations:** 1 R&D, Cellular Technology Ltd., Shaker Hts., OH, USA

## Introduction

Detection of antigen-specific CD8 cells relies on the use of peptides that can bind to HLA-Class I molecules. There is extensive knowledge on individual HLA-alleles’ peptide binding requirements and for many antigens immunogenic peptides have been defined. The 32 individual peptides that comprise the CEF peptide pool represent such well-defined peptide determinants for Cytomegalo-, Epstein Barr-, and Flu- virus. We tested 42 healthy human donors on the accuracy of these peptide predictions. For example, will all HLA-A*0201 positive donors who have been infected with one of these viruses show a CD8 cell response to the pre-defined HLA-A*0201-restricted peptide of that virus? If the donor responds, will it be a dominant response, one of several (co-dominant) responses, a weak (subdominant) response, a barely detectable (cryptic) response, or will the peptide not be recognized while responses to other peptides of the virus prevail? How many times are unpredicted peptides of the virus recognized in a dominant fashion? To the practical end, we asked, whether reliance on select “immunodominant” peptides is a reliable alternative to agnostic immune monitoring with peptide pools.

## Methods

Forty-two HLA-class I, high-resolution-typed, healthy human donors were selected from the CTL ePBMC® library. The PBMC were tested for reactivity to the individual CEF peptides measuring IFN-γ with the ELISPOT assay. To assure low background, serum-free, CTL-Test™ Medium was used. The spots were counted using an ImmunoSpot® S5 Core reader. The predicted vs. the actually detected response was compared.

## Results

Of the expected 241 recall responses, the 32 individual CEF peptides induced a total of 122 positive responses in the 42 donors. Within these 122 positive responses, 36 (30%) were dominant, 41 (34%) were subdominant, and 45 (37%) cryptic. In 119 instances, the predicted peptide was not targeted by CD8 cells detectably. Twenty unpredicted peptides were immune dominant (35%), in 20 instances (35%) unpredicted peptides were subdominant, and in 17 (30%) such peptides elicited weaker, cryptic responses.

## Conclusions

The data clearly shows that predicted peptides are not necessarily immune dominant. In 49% of the test cases, the predicted peptide did not induce a detectable recall response. When it did, it was one of several targeted determinants among which it was subdominant or cryptic. Thus, reliance on one or a few peptides is likely to miss the majority of the antigen-specific CD8 cells, strongly arguing for the use of peptide pools for immune monitoring.

